# Complete genome sequence of *Geobacter* sp. strain 60473, an electrochemically active bacterium isolated from lakeshore mud

**DOI:** 10.1128/mra.01079-23

**Published:** 2023-12-22

**Authors:** Tomoka Harada, Yohei Yamada, Atsushi Kouzuma, Kazuya Watanabe

**Affiliations:** 1 School of Life Sciences, Tokyo University of Pharmacy and Life Sciences, Hachioji, Tokyo, Japan; 2 Seiko Epson Corporation, Fujimi-cho, Suwa-gun, Nagano, Japan; DOE Joint Genome Institute, Berkeley, California, USA

**Keywords:** bioelectrochemistry, microbial fuel cell, microbial electrolysis cell

## Abstract

*Geobacter* sp. strain 60473 is an electrochemically active bacterium (EAB) isolated from mud taken from the shore of lake Suwa in Japan. Here, we report the complete genome sequence of strain 60473, which helps deepen our understanding of common and strain-specific genomic features of EAB affiliated with the genus *Geobacter*.

## ANNOUNCEMENT

Electrochemically active bacteria (EAB) are the focus of recent research for their application to a variety of biotechnology processes, including microbial fuel cells (MFCs), microbial electrolysis cells, and microbial electrosynthesis processes (1). Attempts have been made to isolate novel EAB from the natural environment, some of which exhibit high electrochemical activities (2). In our laboratory, a sediment MFC (sMFC) was operated using mud taken from the shore of lake Suwa (36°03′ N 138°04′ E, Okaya, Nagano, Japan), and after a 2-month operation, EAB were isolated from the anode of sMFC using agar plates containing DSM 826 medium (German Collection of Microorganisms and Cell Cultures GmbH). One of the isolates, named strain 60473, attains relatively high current densities, such as over 1 mA cm^−2^ per projected area of anode, in bioelectrochemical cells (BESs) containing fumarate-free DSM 826 medium. In a BES, a piece of graphite felt served as a working electrode, and a platinum wire served as a counter electrode. The working electrode was poised at −0.2 V vs an Ag/AgCl reference electrode. We are, therefore, interested in its genomic features and determined the complete genome sequence.

Genomic DNA was extracted from an anaerobic culture of strain 60473 grown in DSM 826 medium using FavorPrep Tissue genomic DNA extraction kit (Favorgen, Ping Tung, Taiwan). DNA was fragmented using g-tube (Covaris, Woburn, MA), and 10- to 20-kbp fragments were used to construct a genomic library using SMRTbell Express Template Prep kit 2.0 (Pacific Biosciences, San Diego, CA). After polymerization using Binding kit 2.2 (Pacific Bioscience), it was sequenced using Sequel IIe (Pacific Biosciences). From 19,198 concensus (CCS) reads, HiFi reads were prepared using SMRT Link (v. 11.0.0.146107, Pacific Biosciences) and Filtlong (https://github.com/rrwick/Filtlong), and they were assembled into contigs using Flye ver. 2.9.1-b1780 (3) and Bandage ver. 0.8.1 (4). The completeness of the assembled genome was checked using CheckM ver. 1.2.2 (5), and coding sequences were predicted using Prokka ver. 1.14.5 (6). Default parameters were used for all software unless otherwise specified.

The genomic analysis generated a single contig of 3,793,644 bp in length with a GC content of 61.1%. No plasmid was found. The chromosome contains 2 rRNA operons, 50 tRNA genes, and 3,413 protein-coding sequences. Analyses of the 16S rRNA gene sequence using Blast in the NCBI database (7) indicate that strain 60473 is affiliated with the genus *Geobacter* and closely related to *Geobacter sulfurreducens*. Comparison in global genomic features among *Geobacter* EAB for which complete genomes are available, including, *G. sulfurreducens* strains PCA (CP078092), KN400 (CP002031), YM18 (AP017912), and strain 60473, is shown in Table 1. Detailed analyses of their genomic features, e.g., identification of genes specifically present or absent in respective strains, will help deepen our understanding of common and strain-specific genomic features of EAB affiliated with the genus *Geobacter*.

**TABLE 1:** Comparison in genomic features of strain 60473 and other EAB affiliated with *Geobacter sulfurreducens*

	60473	PCA^*a*^	KN400^*a*^	YM18^*a*^
Genome size (bp)	3,793,644	3,814,128	3,714,272	3,726,411
CDS	3,429	3,711	3,316	3,316
G + C content (%)	61.1	60.5	61.3	60.7
rRNA operon	2	2	2	2
Plasmid	0	0	0	0

^*a*^ These sequences were retrieved from the NCBI database.

## Data Availability

The raw reads are available in the DDBJ Sequence Read Archive (DRA) under the accession number of DRA017481. The complete genome sequence of *Geobacter* sp. strain 60473 was deposited in GenBank/DDBJ under accession number AP028967.
